# Mental State of the Persecuted

**Published:** 1881-02

**Authors:** 


### Original Translations.

MENTAL STATE OF THE PERSECUTED. From *Le Praticien*, Sept. 13, 1880. Translated by H. D. Valin, M.D.

Till late years, persecuted lunatics remained unobserved, and the clinical history of their persecutions was unknown. This was owing to a lack of classification of mental diseases; they were referred to the melancholy of Pinel, the lypæmania of Esquirol, or to the monomania with sad ideas of Baillarger who touched this question, thirty years ago, and divided the monomaniacs into two classes, the gay and the sad.

In 1852, Lasègue described, in the *Archives Générales de Médecine*, a category of patients who came under his observation at the Prefecture of Police. Ideas of persecution were prominent in their disease, and suggested the appropriate name of delirium of persecution. Legrand du Saulle, since 1864, studied the subject in connection with Lasègue among the patients of the Prefecture of Police, wherein every day brings some case of this kind. In 1871 he published a monograph on the delirium of persecution. This delirium is very frequent, since 500 cases occur every year in Paris.

Men predisposed to the delirium of persecution are of little intelligence; the start is slow, and the first cause can be referred sometimes to twenty years distance. No one could observe the evolution of that disease. The subject just attacked, is undecided, foggy, anxious, selfish, and morose; he abstains from the commerce of other people, and lives retired.

This period of evolution, unobserved, except by the patient's relatives, may be slow, and meanwhile he reasons with thought; he coldly accepts humiliation and contrariety without any reaction of his mind. These ideas return after he is calmed, and again he makes up his mind to follow them. Some day he chances to be more bothered than usual; after interrogating himself, he sees hidden enemies, secret strategems; he imagines occult influences, the police, Jesuits, and Free Masons; then physical causes; he accuses nature, invokes electricity, somnambulism, and ventriloquy, imagining himself persecuted by trifling objects. A skeptic at first, his doubts weaken, and the probable becomes a certitude. Then he ceases to dispute, accepts everything, is suspicious of nobody, but accuses the oddest influences. He may take sudden determinations, give up his occupation, change his residence, and accept a lower position because he is mocked, or because he heard somebody whispering about him.

The most important symptoms are hallucinations of hearing; and no decision or determination but he will take to escape them. He will do anything to avoid a return of that hallucination. Accordingly, having formed the habit of retiring from the café at 11, he goes out at 10 since he heard some one insulting him, and then, ceasing to hear anything but insults, he returns, and determines to retire at five. Just now a true influence is felt, that of the medium in which he lives. The linguist has this advantage that his hallucinations are in any language, in French at Paris, in Italian in Milan, etc. A mere change of places often suffices to cause these hallucinations to disappear, but they return.

The original cause of the delirium of persecution often arises from a mere pretext, while the greatest misfortunes produce nothing but prostration and dejection. The persecuted remarks that people stare at him, but generally he has no hallucination of sight, unless he should be an epileptic or a subject of alcoholism. One experiencing abnormal sensations has illusions at first; it seems to him that one passing by whispered his name; a look, exchanged between two persons near him, seems a signal. It is as yet an auditory illusion, yet it strikes him vividly. This period of illusion is succeeded by one of hallucinations; he is very wretched; the threatening voice comes from the ceiling, the floor, the walls, or from his bowels. He claims to have been abused and wronged. He pays attention to all the invectives which he heard through the ceiling, or the floor; he accepts everything, lives by himself, reports everything to himself. He takes no interest in important events, and cares for nothing else but what is done to him. A complete stranger to everything but himself, a more selfish man never was. He does not try to investigate the motives for which he is persecuted, nor the authors of such strategems. A noise strikes his ear; no use to try his sight, it is another trick against him. He abandons himself to fanciful appreciations and imaginary fears. The husband, the father and the citizen have disappeared and left a doubly selfish man, living in his delirious conceit. At last he believes that every one is acquainted with the persecutions which pursue him.

These patients have a peculiar language; it is a sort of special vocabulary. They say that they have been consulted in their thoughts, some one has repeated through their mouth, they allude to their prattlers, to their good and evil voices, but they do not say voices, they say, my invisible, my secret persons, my locations, my speaking ideas, etc. They borrow physical terms, think themselves under the influence of some natural force, and talk of coils, currents, and commotions. Often the hallucination of hearing rests on a true fact. A man who stole a silver spoon, when 18 years old, heard, 25 years later, on entering the asylum, a voice saying: That spoon, you stole it.

One of the most interesting cases is that of Jean Martin, quoted in 1877 by Legrand du Saulle. He heard a voice bringing a real fact back to his memory after thirty-two years: "Here is the masturbator of Father Michel." He was forty-seven then, and he armed himself, bought a revolver, and had a tin coat-of-mail made. His hallucinations were not constant and would cease during Mass from his content of mind. On October 9, 1876, he met the famous Michel, and fired five times at him. After the accomplishment of the murder he calmly delivered himself a prisoner. Three hours later, he wrote a relation of his delirium of persecution to his preceptor. He claimed to have been the victim of Michel for 32 years; the persecuted always inverts rôles. On being confronted with his victim, Jean Martin exclaimed, "I recognize here the rotten being who poisoned my life; he has been my murderer." From that time, hallucinations of hearing ceased for eighteen months, in Meoulin's Asylum, where he was placed, but returned under another form; now the guillotine threatens him.

The persecuted have associated ideas with fundamental ones. They are hypochondriac, and liable to murder their physicians. They dread to be poisoned, hence their life is unbearable. The persecuted buys his food himself, never deals twice with the same man, and may even leave the country on that pretext. They try to poison him with unseen powders or gases. Powders are thrown into his room, or they come down the chimney into his bed, hence he goes to sleep elsewhere. He speaks of asphyxiating gases (for he seeks no serious explanation of things), putrefied miasms, and imponderous chemical atmospheres. These gases fall from the ceiling, along the chimney, or escape through the floor; then he closes all the openings, else he leaves the windows open at night in winter.

As an associated delirious conception, there is also the idea of greatness. They infer that the will which leads such unremittent enemies must be very stubborn, and their number great. Some day they conclude that they must be important men to excite so much animosity. They study their person and ask, who am I? But there are many bastards in that class, which fact lowers their characters and their manners, for such a position is ill-born in life. After meditating, the persecuted believes that he may be the son of an important man, then writes his novel; he was substituted for another child; he is the son of Don Carlos, or of Napoleon III. After the delirious theory is formed, they never get over it. They may even bring suits before the law, and may find proofs to substantiate their claims.

How frequent is the delirium of persecution? Lasègue and Legrand du Saulle found one in every six or seven insane. It is most common in men from the age of 30 to 40 years, the time of great works and excesses; in women, from 40 to 50. It has been remarked that celibacy and widowhood are most frequent causes. It is difficult to determine the influence of hereditary descent, for insanity is not readily divulged by the relatives because compromising and dishonoring. Legrand du Saulle remarked that a great share is owing to inheritance from the mother, which is to that from the father in the proportion of three-fifths to two-fifths.

There is a multiplicity of causes, and it is difficult to affirm that the disease depends on any one in particular; lasting sorrows, seminal losses, and syphilis, are prominent causes. The depressing influence left in a predisposed mind by the idea of having taken mercury may be remarked. The delirium may point to some real fact as its origin. The course is slow, and the incubation is not easily determined. Only 20 per cent. recover. These at first experience slight intermissions or a temporary relief. Some are absolutely timid, but they are reserved and dissimulate; hence one should be very cautious. They well know that they are under the influence of a hallucination, but they wish to be discharged, and dissimulate their disease. The perpetual fear to be taken for masturbators is a torment of their life. Some persecuted institute law suits, or refer to the State, the rulers, or the government ministers, to obtain justice; some have walked to Paris from great distances for that purpose.

When the persecuted makes threats, it is because he is active. He always walks forward and kills. The passive persecuted is he that kills himself.

Taking other criminals and insane into consideration, the persecuted are divided into three groups: 1. Those that are not dangerous; 2. Those that are dangerous to themselves, not others; 3. Those dangerous to others. The first category is numerous enough. The second class comprises those who kill themselves. The third class is very dangerous; it comprises the numerous insane who commit crimes. A man sawed his wife's neck, because he heard a secret voice telling him that she had deceived him with somnambulists.

Various persecuted succeed in imparting their raving conviction to some acquaintance whom they render as insane as themselves, but much intimacy is necessary for that pathological contagion, as husband and wife, mother and daughter. This Lasègue lately described as double insanity. One can see these two persons raving in the same manner; but one may be capable of being cured, the passive, and the active incurable. If the passive is removed from his companion, he may recover. He will lose his convictions, while the active remains firm in the delirium created by himself. But what is peculiar in that fact, is that the passive hears the same hallucinations, and the same same gastric voices; he is a victim to the same sensorial errors.

Ideas of persecution are met in the aged, as also in subacute alcoholism sometimes. The persecuted are difficult to treat because they dread to be poisoned. Some have recourse to hydrotherapy, which is capable of exciting or appeasing them. Leuret erected moral culture into a systematic treatment. The physical condition and its tendencies must be treated.

As a rule the persecuted strikes only the individual whom he has forewarned; this is peculiar to him. They make many wills, and forget their family; give to the poor, to the hospital, to any body; some leave their fortune to the Quinze-Vingt. It is difficult to determine their degree of responsibility; but it is always necessary to know whether the persecuted had hallucinations. As soon as hallucinations have had their sway, reason and free-will are lost, man is no longer his own master, and is no longer responsible.

---

THE annual reunion and banquet of the Alumni Association of Rush Medical College will be held in the Grand Pacific Hotel of this city, on the evening of Feb. 22d. The commencement exercises of the college will be held in Central Music Hall on the afternoon of the same day. The commencement exercises of the Chicago Medical College and the annual banquet and reunion of its alumni will be held on Tuesday, March 29th; due notice of the place will be given in our next number. The practitioners' courses of both schools will begin in and continue through the month of April.

